# President Foster’s Address

**Published:** 1890-10

**Authors:** 


### Editorial.

### PRESIDENT FOSTER'S ADDRESS.

THE importance of the questions discussed in the address before the American Dental Association at Excelsior Springs, Missouri, can scarcely be overestimated, and, coming as they do from the official head of the representative association of the United States, they should receive careful consideration. It is unusual, perhaps, for the president of the association to discuss questions of this character; but if it is novel, it furnishes a most excellent precedent, and one worthy to be followed by those who may succeed him. The influence of his broad outlook over a series of subjects most interesting at the present time must be widely felt, and should be thoughtfully pondered.

The question he prominently desired to be considered is the necessity for the unification of the laws regulating dentistry, and his arguments based on the present chaotic condition of these enactments, it seems to us, should have special consideration by those most intimately interested.

It is not surprising that the crudities of legislation in this direction should have resulted, as a whole, in a mass of ill-considered statutes. This was the natural result of inexperience. Law is the outgrowth of necessity. It is an evolution from the extreme liberty, bordering on license, on the one hand, to an organized effort at restraint on the other. It is the child grasping at the meaning of mature ideas, but incapable of formulating them into connective thought.

The older professions in this country have managed for long periods to develop under the force of a public opinion that demanded the highest culture attainable; but this, powerful as it has been, has had only a moderate degree of success. Dentistry has not had until very recently this moral force behind it. The public have been slow to believe that it was more than a trade and was amenable, as ordinary business, to the laws of demand and supply. Hence there seemed to be a necessity for force measures, more imperative, possibly, in dentistry than in medicine. The line had to be sharply drawn somewhere, and the various State organizations began the work by petitioning the legislatures for relief by the enactment of certain laws for the government of dental practice. It may be considered remarkable that, under the circumstances, the work has been as well done as we find it in the statute books.

The question of bringing law to bear on a body such as ours has been seriously questioned by some of the ablest men in our ranks. It is one difficult of solution, and time can alone determine whether the result is to be for the good or ill of dentistry in this country. It is conceded that the condition a few years back, when the American degree in dentistry was almost universally discredited, was ample justification for active measures looking to the entire eradication of the evil influences that were sapping the very life of our profession.

With characteristic energy the dentists of the United States spontaneously resolved to effect a change, and in a comparatively short period their influence was powerful enough to have laws adopted in nearly every State in the Union. This was rapidly followed by State Boards of Examiners, and subsequently was formed the National Association of Examiners, a delegated body deriving its power from the several State Boards. Having passed through this period of organization we are about to enter a new phase of this movement, which will require the best wisdom to carry to a successful issue. Law is a weapon which carries a double edge and may react to the injury of those who use it.

The present condition of the various statutes is very far from satisfactory, and the operation of these laws has, in many instances, worked serious injury to individuals. The reasons for this have been well and ably portrayed by President Foster, and need not be repeated here. If we must have law, let it bear directly upon the wrong committed. Let it press hard, if necessary, upon the individual who has made no effort at culture and mildly on the student who has made every effort to secure the best knowledge attainable. This view, unfortunately, has not prevailed in some of the States; indeed, it would seem as though the uppermost thought was one of sympathy with the one who has made little or no effort, while all the legal force is heavily arrayed against the young man who has devoted years, it may be,—and at great expense and labor,—to master the difficulties of his profession. That this is wrong requires no argument, and is an injustice so palpable that it is surprising that the intelligent members of State organizations should have ever entertained such an imperfect idea of what was needed at that critical period. It is, however, embodied into law, and State Boards are to-day granting degrees, or what is equivalent to them, while graduates of colleges are required to be re-examined, and in some States are forced to pay a heavy additional fee for the privilege.

The examinations of these boards might not be so serious, indeed would have a certain value, were it not for the fact that these bodies are irresponsible. The profession has no guarantee that these men are qualified for the positions to which they have been appointed. The ability to examine is not easily acquired, and it may be a question whether it is not an inherited talent and does not belong to every one. Whether this be true or not, it needs a large degree of experience to carry it forward successfully. If the present form of State Examining Boards is to remain as part of the educational work, then a National Body composed of distinguished specialists should be selected to examine the men proposed for these responsible positions. When this is done there will be some degree of confidence in the result.

The subject is too extended in its ramifications to be commented on here with the fulness required. It needs time and space commensurate with its importance, which are not at our command in this issue of the JOURNAL.

The American people should ever be alert and jealous of any infringement of their individual rights. Law is of slow growth, but is proportionately insidious, and will, in time, destroy the best organized republic if allowed to have undisputed sway. The people of the Old World are supposed to be suffering from the tyranny of despotic power. While this may be true in a limited degree, they are more the victims of laws and customs, the growth of centuries. It has, therefore, been the opinion of many of those best informed that a republic could exist only in name in what is known as European governments. The overturning of a throne means, therefore, if this view be correct, no change in the direct controlling force used to influence these oppressed peoples, for the simple reason that the laws have become part of their very existence, and the fall of dynasties can have no effect upon them. Should we not, then, approach all attempts at formulating law with this query: Is this for the good of the public? If so, work for its adoption. If, on the other hand, it means oppression and selfish exclusion of others from the special work we are engaged in, would it not be well to hesitate and refuse to have part or lot in manacling the profession?

